# Supplementary material for: Researchers and Their Experimental Models: A Pilot Survey in the Context of the European Union Health and Life Science Research
Source: Animals (Basel). 2022 Oct 14;12(20):2778. doi: 10.3390/ani12202778 (PMC9597815; doi:10.3390/ani12202778)

SUPPLEMENTARY TABLES

Supplementary Table S1. Other international surveys in the Three Rs field or among researchers are shown.

Survey Name	Organizer	Participants	Year
Scientific methodologies for the assessment of combined effects of chemicals.	EC-JRC	58	2015
3Rs knowledge sharing.	EC-JRC	351	2017
Biologically based mathematical models in toxicology: current use and regulatory acceptance.	EC-JRC	93	2017
Establishing the scientific validity of complex in vitro models.	EC-JRC	646	2018
A survey on Monitoring Innovation and Societal Impact of EU-funded Research.	EC-JRC	202	2020
Non-animal derived antibodies.	EARA/EFPIA	133	2020
Publication Bias.	HSI/PCRM	90	2021
Biologicals Manufacturers 3Rs implementation.	NC3Rs	28	2021
	**Average**	200	
	**SD**	207	
	**MEDIAN**	113	

Supplementary Table S2. Health and Life science research fields of the survey participants.

RESEARCH FIELD	%
NEUROSCIENCE	15,6%
TOXICOLOGY	9,9%
ONCOLOGY	5,7%
BIOMEDICINE	5,0%
REGENERATIVE_MEDICINE	4,3%
DRUG_DEVELOPMENT	3,5%
IMMUNOLOGY	3,5%
PULMONOLOGY	3,5%
DEVELOPMENTAL_BIOLOGY	2,1%
MICROBIOLOGY	2,1%
NEPHROLOGY	2,1%
STEM_CELL_BIOLOGY	2,1%
VIROLOGY	2,1%
CARDIOLOGY	2,1%
BIOCHEMISTRY	1,4%
BIOMATERIALS	1,4%
BIOMEDICAL_ENGINEERING	1,4%
CELL_BIOLOGY	1,4%
PHARMACOLOGY	1,4%
PHYSIOLOGY	1,4%
VETERINARY	1,4%
ANIMALS_WELFARE	1,4%
BIO-ENGINEERING	1,4%
LIFE_SCIENCES	1,4%
ORGAN-ON-CHIP	1,4%
3RS	0,7%
ANALYTICAL_BIOSCIENCES	0,7%
AUTOIMMUNITY	0,7%
BIOFABRICATION	0,7%
BIOLOGY_OF_REPRODUCTION	0,7%
BIOPHYSICS	0,7%
BIOTECHNOLOGY	0,7%
CHRONIC_PAIN	0,7%
COMPUTATIONAL	0,7%
DERMATOLOGY	0,7%
FISH_BIOLOGY	0,7%
GASTROENTEROLOGY	0,7%
GENETICS	0,7%
HYPERTENSION	0,7%
INFLAMMATION	0,7%

INFLAMMATORY_SKIN_DISEASE	0,7%
MATHEMATICS	0,7%
METEBOLOMICS	0,7%
MICROFLUIDICS	0,7%
MOLECULAR	0,7%
NANOMEDICINE	0,7%
NON-INVASIVE_IMAGING	0,7%
NUMERICAL_ANALYSIS	0,7%
ORGAN_FIBROSIS	0,7%
ORTHOPEDICS	0,7%
REGULATORY	0,7%
SYSTEM_BIOLOGY	0,7%
VALIDATION	0,7%
VASCULAR_SURGERY	0,7%

SUPPLEMENTARY FIGURES

Supplementary Figure S1. Geographical distribution of the participants in Europe.

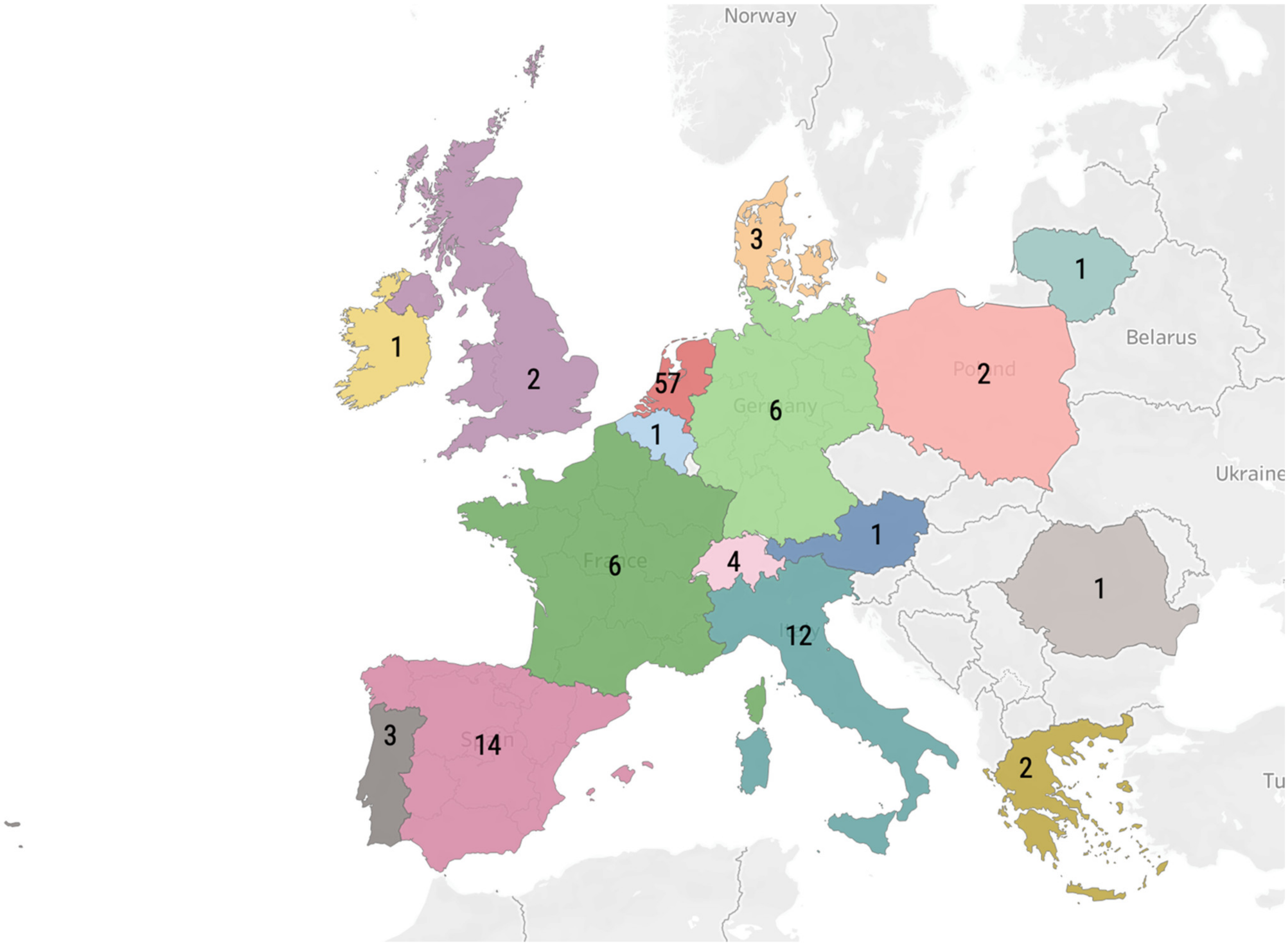

Supplementary Figure S2. Participants answered to the following question "How did you choose or establish your main teaching tool/media?". The bar chart shows the methods used by respondents to choose or establish their teaching media.

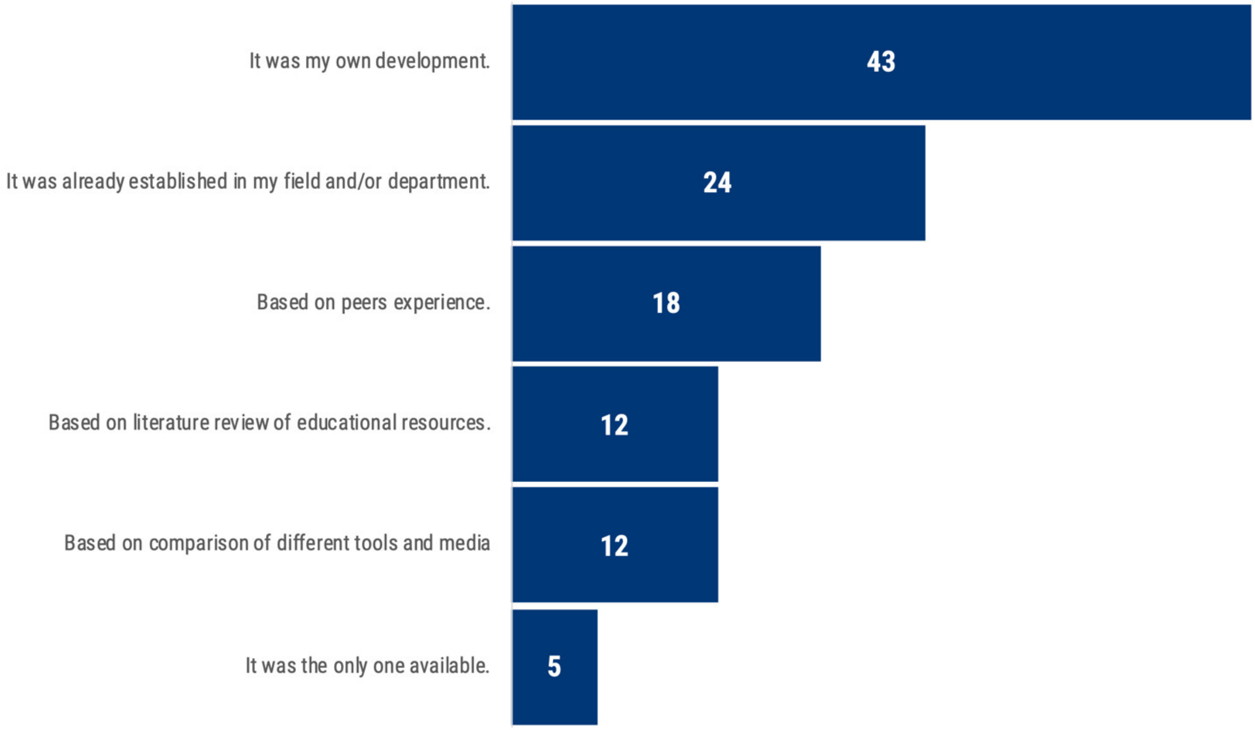

Supplementary Figure S3. Participants answered to the following question "If an animal model and a new non-animal model provide comparable results, do you think the Directive 2010/63/EU acknowledges adequately the animal model by virtue of its well known, predictable, widely used and established?" Answers were segmented by the involvement (dark blue) or not (red) of respondents in regulatory science projects.

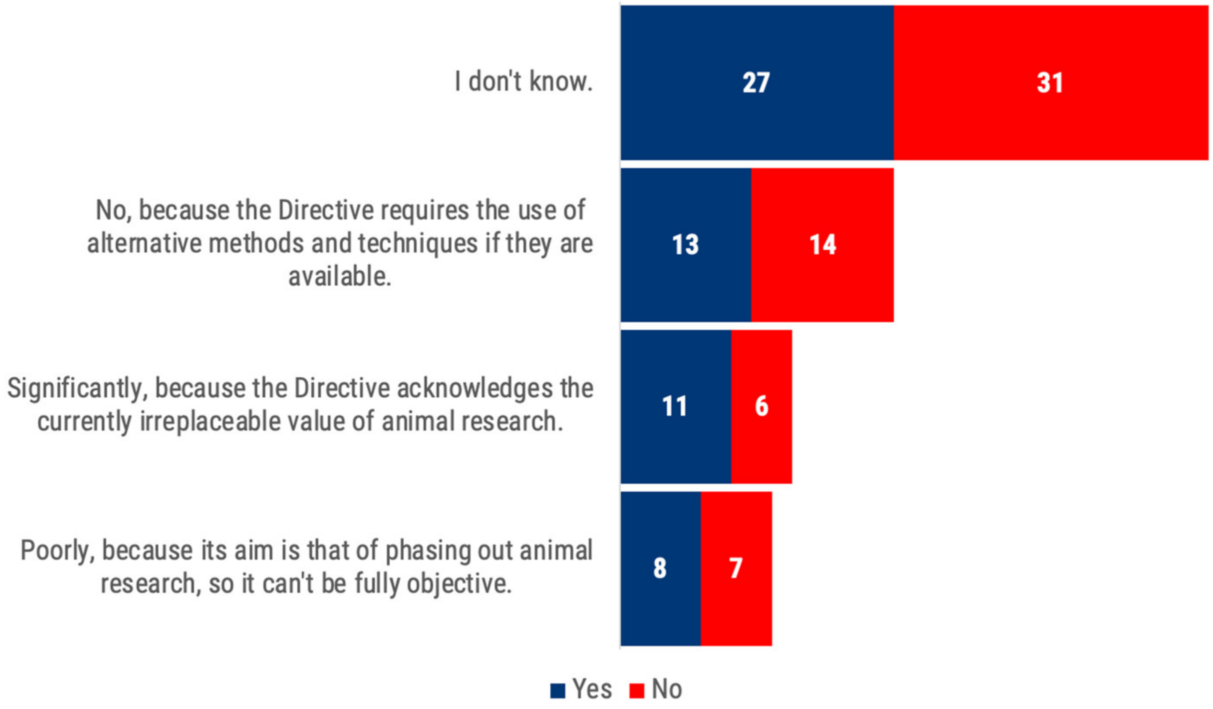

Supplement: Supplementary file 1 [file animals-12-02778-s001.zip › animals-1938813-supplementary.pdf]
